# Supplementary material for: Dual-Task Optimization Method for Inverse Design of RGB Micro-LED Light Collimator
Source: Nanomaterials (Basel). 2025 Jan 25;15(3):190. doi: 10.3390/nano15030190 (PMC11820347; doi:10.3390/nano15030190)
Supplement: Supplementary file 1 [file nanomaterials-15-00190-s001.zip › nanomaterials-3392379-supplementary.pdf]

Supplemental Materials for

Dual task optimization method for inverse design of RGB micro-LED light collimator

Liming Chen [1], Zhuo Li [1], Purui Wang [1], Sihan Wu [1], Wen Li [1], Jiechen Wang [1], Yue Cao [1], Masood Mortazavi [1], Liang Peng [1] and Pingfan Wu [1,*]

[1]Futurewei Technologies, 645 Martinsville Road, Basking Ridge, New Jersey 07920, USA
* Correspondence: pwu2@futurewei.com

Figure S1. Far-field angular power distributions of 20 separate simulations for the blue channel.

Figure S2. Far-field angular power distributions of 20 separate simulations for the green channel.

Figure S3. Far-field angular power distributions of 20 separate simulations for the red channel.

Figure S4. The far-field angular power ratio density distribution for a collimator designed using three incident angles (i.e., -15°, 0°, 15°) in the dual task (i.e., color routing). (a) – (c) power distribution for the blue, green, and red channel. The total output power flux percentage were 29.51%, 25.12% and 24.84%. The power ratio within ± 20° in the far field were 57.42%, 55.67% and 61.93%.

Figure S5. The collimation performance evaluation results of the structure with the fabrication constraint (i.e., smallest feature size was approximately 28.87 nm). (a) The designed structure with fabrication constraint and without fabrication constraint. (b) – (d) The far-field angular power ratio density distribution after using the structure with fabrication constraint of blue, green and red light, respectively. The total output power flux percentage were 28.09%, 22.90% and 24.19%. The power ratio within ± 20° in the far field were 59.47%, 54.00% and 57.97%.

Figure S6. The effect of different β towards the binary projection function. The parameter η is set as 0.5.

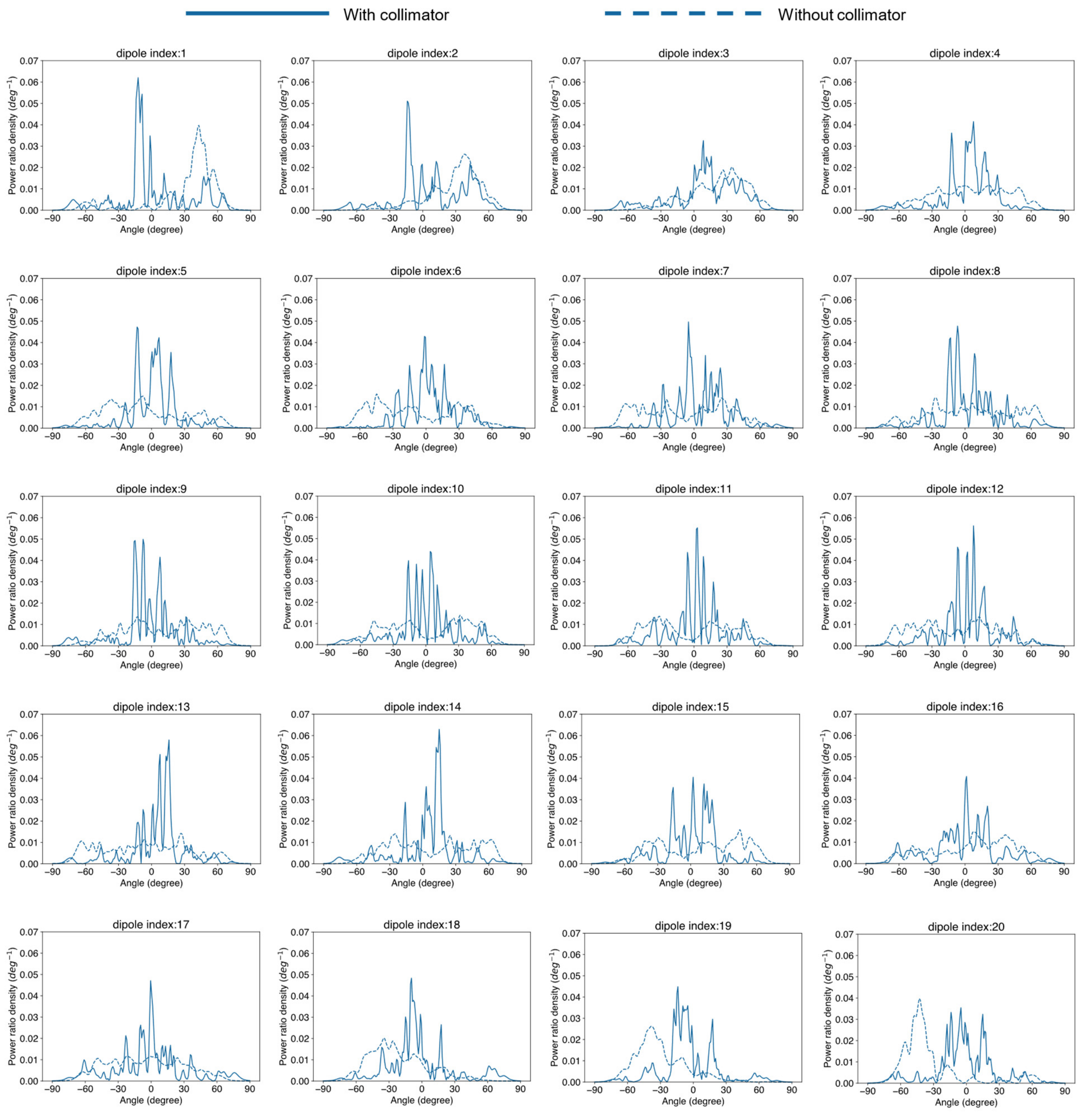

Figure S1. Far-field angular power distributions of 20 separate simulations for the blue channel.

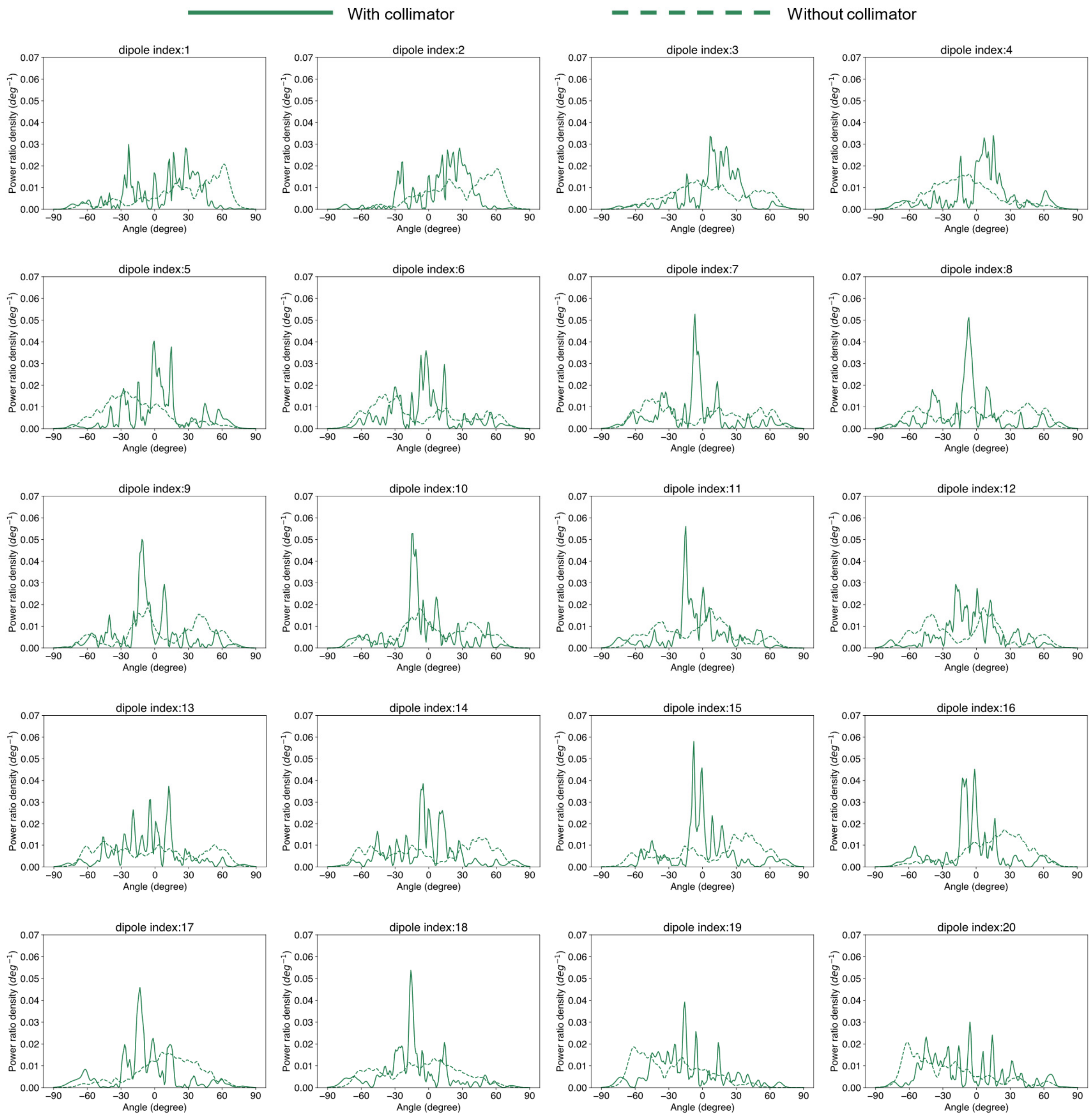

Figure S2. Far-field angular power distributions of 20 separate simulations for the green channel

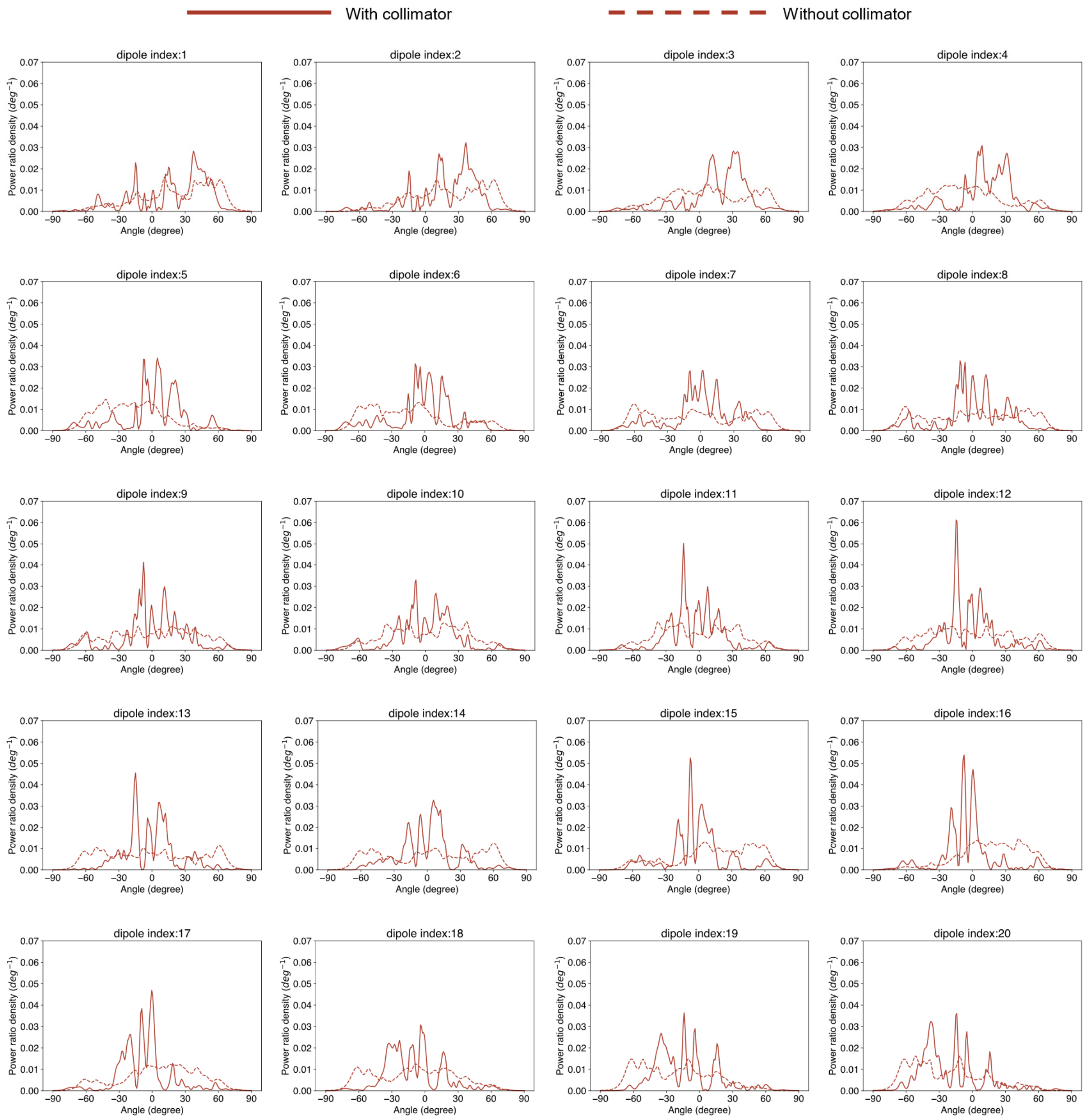

Figure S3. Far-field angular power distributions of 20 separate simulations for the red channel.

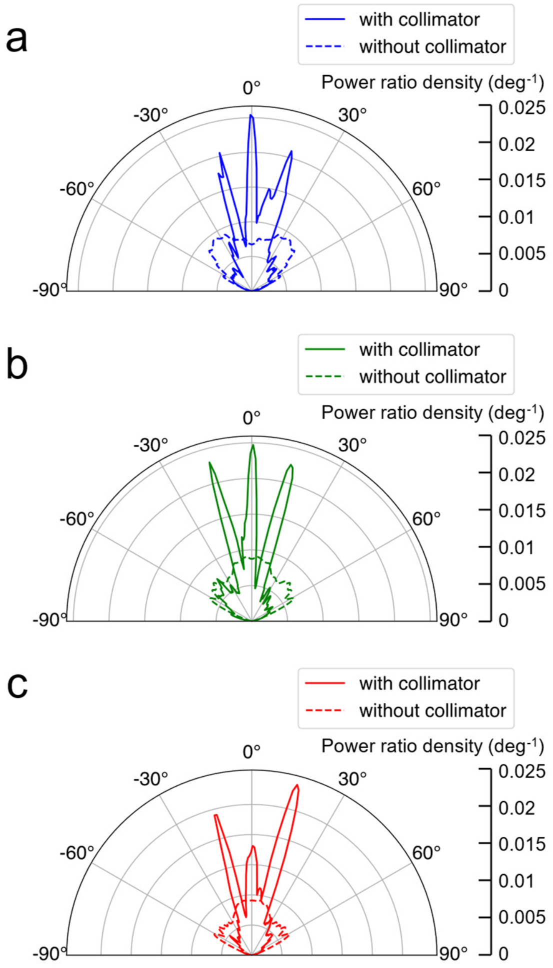

Figure S4. The accumulated far-field angular power distribution for a collimator designed using three incident angles (i.e., -15°, 0°, 15°) in the color routing task. (a) – (c) power distribution for the blue, green, and red channel. The total output power flux percentage were 29.51%, 25.12% and 24.84%. The power ratio within ± 20° in the far field were 57.42%, 55.67% and 61.93%.

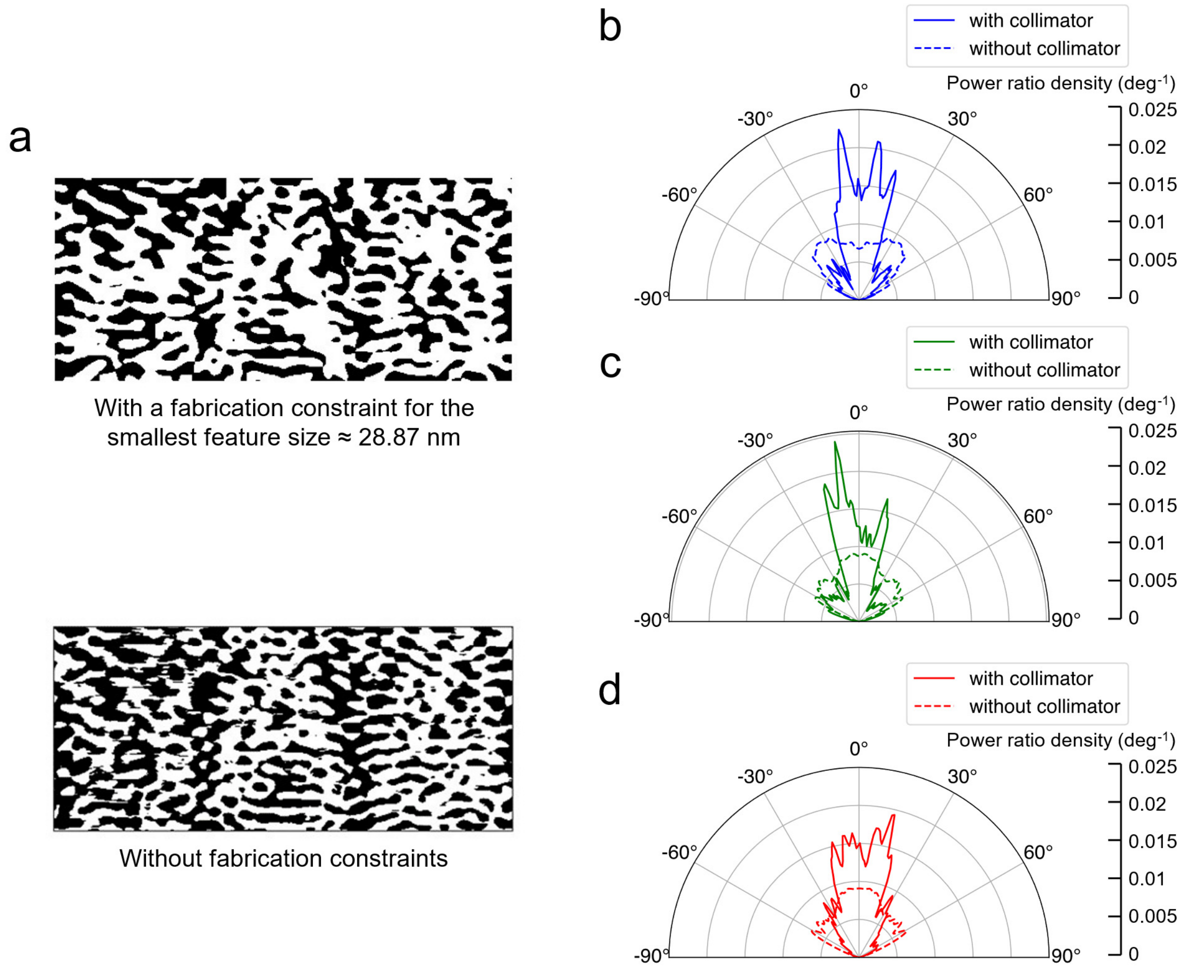

Figure S5. The collimation performance evaluation results of the structure with the fabrication constraint (i.e., smallest feature size was approximately 28.87 nm). (a) The designed structure with fabrication constraint and without fabrication constraint. (b) – (d) The far-field angular power ratio density distribution after using the structure with fabrication constraint of blue, green and red light, respectively. The total output power flux percentage were 28.09%, 22.90% and 24.19%. The power ratio within ± 20° in the far field were 59.47%, 54.00% and 57.97%.

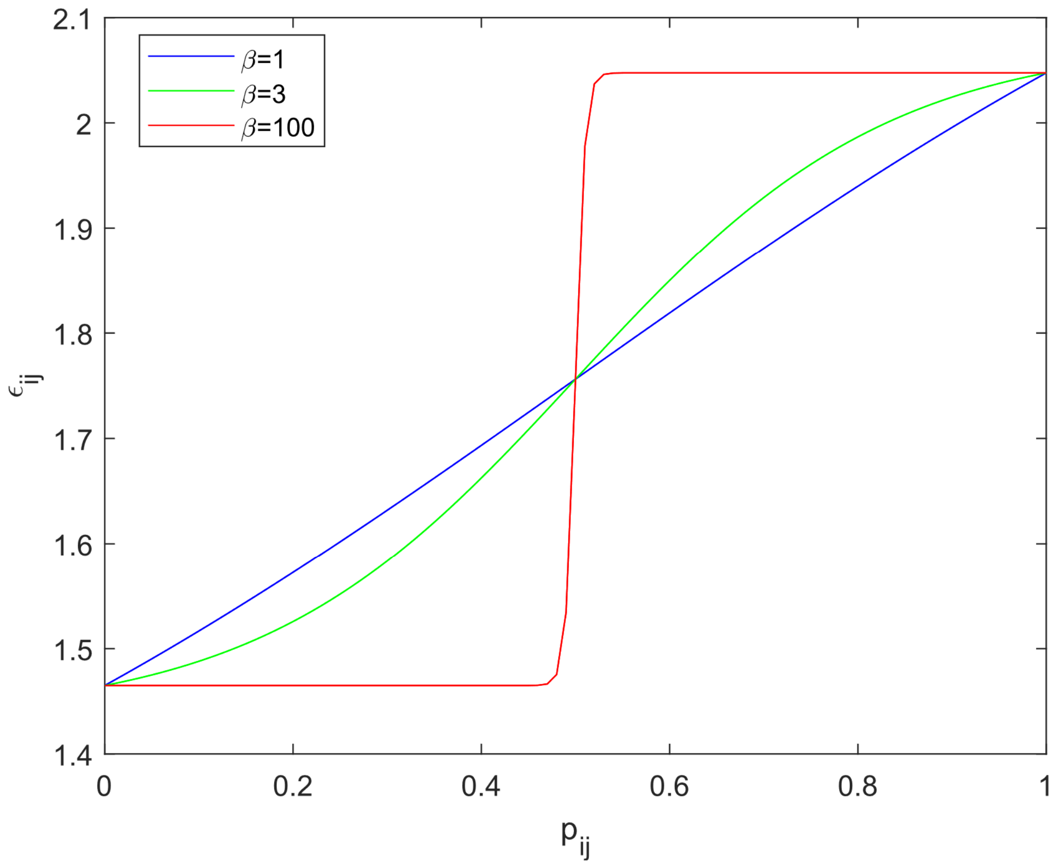

Figure S6. The effect of different β towards the binary projection function. The parameter η is set as 0.5.
